# Two distinct Do-Not-Resuscitate protocols leaving less to the imagination: an observational study using propensity score matching

**DOI:** 10.1186/s12916-014-0146-x

**Published:** 2014-08-29

**Authors:** Yen-Yuan Chen, Nahida H Gordon, Alfred F Connors, Allan Garland, Shan-Chwen Chang, Stuart J Youngner

**Affiliations:** Department of Social Medicine, National Taiwan University College of Medicine, No. 1, Rd. Ren-Ai sec. 1, Taipei, 10051 Taiwan; Department of Medical Education, National Taiwan University Hospital, No. 7, Rd. Chong-Shan S., Taipei, 10002 Taiwan; Department of Bioethics, Case Western Reserve University School of Medicine, 10900 Euclid Avenue, Cleveland, OH 44106-4976 USA; Department of Medicine, Case Western Reserve University School of Medicine at MetroHealth Medical Center, 2500 MetroHealth Drive, Cleveland, OH 44109 USA; Department of Medicine and Community Health Services, University of Manitoba, 66 Chancellors Cir, Winnipeg, Manitoba MB R3T 2N2 Canada; Department of Internal Medicine, National Taiwan University College of Medicine, Address: No. 1, Rd. Ren-Ai sec. 1, Taipei, 10051 Taiwan; Department of Internal Medicine, National Taiwan University Hospital, Address: No. 7, Rd. Chong-Shan S., Taipei, 10002 Taiwan

**Keywords:** Medical care, Cardiopulmonary resuscitation, Do-Not-Resuscitate

## Abstract

**Background:**

Do-Not-Resuscitate (DNR) patients tend to receive less medical care after the order is written. To provide a clearer approach, the Ohio Department of Health adopted the Do-Not-Resuscitate law in 1998, indicating two distinct protocols of DNR orders that allow DNR patients to choose the medical care: DNR Comfort Care (DNRCC), implying DNRCC patients receive only comfort care after the order is written; and DNR Comfort Care-Arrest (DNRCC-Arrest), implying that DNRCC-Arrest patients are eligible to receive aggressive interventions until cardiac or respiratory arrest. The aim of this study was to examine the medical care provided to patients with these two distinct protocols of DNR orders.

**Methods:**

Data were collected from August 2002 to December 2005 at a medical intensive care unit in a university-affiliated teaching hospital. In total, 188 DNRCC-Arrest patients, 88 DNRCC patients, and 2,051 non-DNR patients were included. Propensity score matching using multivariate logistic regression was used to balance the confounding variables between the 188 DNRCC-Arrest and 2,051 non-DNR patients, and between the 88 DNRCC and 2,051 non-DNR patients. The daily cost of intensive care unit (ICU) stay, the daily cost of hospital stay, the daily discretionary cost of ICU stay, six aggressive interventions, and three comfort care measures were used to indicate the medical care patients received. The association of each continuous variable and categorical variable with having a DNR order written was analyzed using Student’s *t*-test and the χ^2^ test, respectively. The six aggressive interventions and three comfort care measures performed before and after the order was initiated were compared using McNemar’s test.

**Results:**

DNRCC patients received significantly fewer aggressive interventions and more comfort care after the order was initiated. By contrast, for DNRCC-Arrest patients, the six aggressive interventions provided were not significantly decreased, but the three comfort care measures were significantly increased after the order was initiated. In addition, the three medical costs were not significantly different between DNRCC and non-DNR patients, or between DNRCC-Arrest and non-DNR patients.

**Conclusions:**

When medical care provided to DNR patients is clearly indicated, healthcare professionals will provide the medical care determined by patient/surrogate decision-makers and healthcare professionals, rather than blindly decreasing medical care.

**Electronic supplementary material:**

The online version of this article (doi:10.1186/s12916-014-0146-x) contains supplementary material, which is available to authorized users.

## Background

When the American Heart Association first approved the clinical use of cardiopulmonary resuscitation (CPR) in 1974, it also proposed that withholding or withdrawing CPR (Do-Not- Resuscitate (DNR) orders) is ethically appropriate if the anticipated benefit outweighs the harm [1]. However, since then, the literal meaning of DNR has not been clear, thus causing confusion that remains problematic in clinical practice.

Several important guidelines have pointed out that DNR precludes only CPR being performed in the event of cardiac or respiratory arrest, and it should not influence care that is medically and ethically appropriate before arrest [2-4]. Nevertheless, several studies have shown that healthcare professionals tended to provide less medical care after DNR orders were written for patients [5-8]. This has raised the concern that at least some DNR patients might be medically or psychologically abandoned [9]. Although these studies did not specifically demonstrate abandonment, they may have reflected misinterpretation by or confusion of healthcare professionals about the proper level of medical care that should be provided to DNR patients [5-8]. Some healthcare professionals may have believed that DNR patients were eligible to receive medical interventions to extend life short of an arrest, while others thought that DNR was, essentially, an order for comfort care only. The latter interpretation of medical care provided to DNR patients has raised the ethical concern that healthcare professionals may blindly decrease medical care if patients do have a DNR order.

These findings have also raised the prospect that some patients who chose DNR because they were ready to die and wanted comfort care only were being treated more aggressively than they would have wanted. Conversely, other patients may have chosen DNR because although CPR offered little hope in the event of a cardiopulmonary arrest, other aggressive interventions, such as cardioversion or even mechanical ventilation, had a reasonable chance in cases short of arrest of returning the patient home to an acceptable quality of life. For the latter group of DNR patients, comfort care only would have been entirely inappropriate.

To provide a clearer and more consistent approach for DNR patients, the Ohio Department of Health adopted a Do-Not-Resuscitate law in 1998, indicating two distinct DNR protocols of DNR orders that allow DNR patients to choose the medical care they want. The first was named Do-Not-Resuscitate Comfort Care-Arrest (DNRCC-Arrest), indicating that DNRCC-Arrest patients are eligible to receive aggressive interventions to extend life until the moment of cardiac or respiratory arrest. The second, named Do-Not-Resuscitate Comfort Care (DNRCC), directed that DNRCC patients should receive only comfort care after the order is written [10,11].

In accordance with Ohio’s Do-Not-Resuscitate law, decisions about DNRCC or DNRCC-Arrest have to be made by the patients, or surrogate decision-makers for incompetent patients, in consultation with their physicians. Our study aimed to examine the medical costs for DNRCC, DNRCC-Arrest or non-DNR patients, as well as the aggressive interventions and comfort care measures they received. The hypotheses of this study were as follows: 1) medical care provided to DNRCC or DNRCC-Arrest patients is not less than that provided to non-DNR patients; 2) DNRCC patients do not receive less medical care after the order is initiated; and 3) DNRCC-Arrest patients do not receive less medical care after the order is initiated.

## Methods

This study was approved by the Institutional Review Board at MetroHealth Medical Center (IRB07-01218).

### Data collection

The data were collected in the medical intensive care unit (ICU) at MetroHealth Medical Center, an urban, 520-bed, and university-affiliated tertiary teaching hospital located in West Cleveland, Ohio. The ICU is a 13-bed, closed-model ICU, where medical care for all patients is shared by nine intensivists. Each intensivist performs two-week blocks of time, generally with weekend cross-coverage by a different intensivist.

The cohort for this observational study was concurrently and retrospectively collected from August 2002 to December 2005 (excluding March to May in 2004, during which time data collection was suspended due to personnel limitations). Only initial admissions to the ICU were included. Data were collected on patients with DNRCC orders, DNRCC-Arrest orders and non-DNR decisions during their ICU stay were collected. Patients were excluded if they changed the DNR order during their ICU stay, either from DNRCC-Arrest to DNRCC, or from DNRCC to DNRCC-Arrest. We also collected patient demographics and clinical data.

Severity of illness on ICU admission was assessed using the presence of each of the pre-existing 30 Elixhauser comorbidity measures [12], Glasgow Coma Scale (GCS), and the total points of the (Acute Physiology and Chronic Health Evaluation (APACHE) II score [13] minus the total points of the GCS. The ICU admission diagnosis was initially recorded based on the 50 APACHE II diagnostic categories [13], then the 50 categories were collapsed to five categories: medical–respiratory, medical–gastrointestinal, medical–cardiovascular, medical–neurological, and others.

### Medical care

We used six aggressive interventions and three comfort care measures to represent the medical care provided to patients. The six aggressive interventions included pharmacological/electrical cardioversion, vasopressor, intravenous antibiotics, renal replacement therapy, blood component transfusion, and central venous line placement. The three comfort care measures included pain control using morphine/fentanyl, pastoral care, and hospice/palliative care consultation. We recorded whether the six aggressive interventions and three comfort care measures were provided to the patients during their ICU stay. In addition, we recorded the six aggressive interventions and three comfort care measures provided to DNRCC and DNRCC-Arrest patients before and after the orders were put into effect.

To partly reflect the medical care that patients received, we also examined the daily cost of ICU stay, the daily cost of hospital stay, and the daily discretionary cost of ICU stay for DNRCC, DNRCC-Arrest and non-DNR patients. “Discretionary cost” was defined as cost including pharmacy, radiology, laboratories, blood bank, and echocardiography [14].

### Propensity score matching

The results of randomized controlled trials (RCT) provide the most rigorous evidence to support the relationship between the independent variable of interest and the dependent variable. However, because of ethical concerns, this ideal study design is usually difficult to attain, particularly in an observational study. Unlike in an RCT, researchers in observational studies do not have any control over the treatment assignments. Thus, confounding variables may have very different distributions between the treatment group and the control group. Matching members of the treatment group to members of the control group by confounding variables is used in observational studies to reduce bias and approximate the design of a RCT [15,16].

To compare the medical care provided to DNRCC patients with that to non-DNR patients, and the medical care provided to DNRCC-Arrest patients with that to non-DNR patients, we established two propensity score models to control for confounding variables using multivariate logistic regression: Model 1 was for DNRCC and non-DNR patients, while Model 2 was for DNRCC-Arrest and non-DNR patients. Each model was built using a non-parsimonious cluster of confounding variables, including patient demographics and clinical data. We did not include the six aggressive interventions and three comfort care measures as the confounding variables in the propensity score models. Whether a DNRCC (or DNRCC-Arrest) order was written during the ICU stay was the dependent variable of Model 1 (or Model 2). Each patient’s propensity score of having a DNRCC (Model 1) or DNRCC-Arrest (Model 2) order written during their ICU stay was obtained.

Greedy matching based on propensity scores was conducted [17]. In Model 1, a DNRCC patient was matched to a non-DNR patient with the nearest propensity score without a caliber. In Model 2, a DNRCC-Arrest patient was matched to a non-DNR patient with the nearest propensity score without a caliber. The confounding variables in the propensity score model were expected to be balanced between the DNRCC and non-DNR groups, and between the DNRCC-Arrest and non-DNR groups. The relationships between having a DNRCC (or DNRCC-Arrest) order written and the medical care were directly examined.

If any of the confounding variables were not balanced in the propensity score models, further multivariate linear regression analysis or multivariate logistic regression analysis was used to control the unbalanced confounding variable, the propensity score, and the DNR decision, depending on the scale of the dependent variable.

### Sensitivity analysis

One limitation of examining the association between an independent variable and the dependent variable using propensity scores matching is that important confounding variables are not adjusted for in the propensity score model. The propensity score of each subject may be seriously degraded if influencing confounding variables are not included in the propensity score model [18].

We assumed that confounding variables that should be included in a propensity score model correlate with each other. A confounding variable is at some level represented by other confounding variables included in a propensity score model. Therefore, an influencing confounding variable, if not included in a propensity score model, does not hurt the propensity score model very much because it is partly represented by other confounding variables being included in the model. According to this assumption, we determined how sensitive the two propensity score models are by removing one confounding variable, age. We then tested the association between DNR decisions and the daily cost of ICU stay, the daily cost of hospital stay, and the daily discretionary cost of ICU stay after controlling for confounding variables excluding age.

### Statistical analysis

We summarized all variables using frequency distributions for categorical variables, and measures of central tendency (mean ± standard deviation) for continuous variables. The association of each continuous variable and categorical variable with having a DNR order written was analyzed using Student’s *t*-test and the χ^2^ test, respectively. Whether each confounding variable was balanced was examined using Student’s *t*-test/χ^2^ test and standardized difference [19]. The six aggressive interventions and three comfort care measures before and after the DNRCC/DNRCC-Arrest order was initiated were compared using McNemar’s test. All statistical analyses were executed using STATA/MP (V11.0 for Windows).

## Results

A sample of 2,440 patients was collected; of these, 2,051 were non-DNR patients and the remaining 389 (15.94%) had DNR orders. Of these 389 patients, 188 had only a DNRCC-Arrest order written and 88 patients had only a DNRCC order written during their ICU stay. 

Before controlling for the confounding variables, DNRCC and DNRCC-Arrest patients were older than non-DNR patients, and had more severe clinical conditions. The daily cost of ICU stay, daily cost of hospital stay, and daily discretionary cost of ICU stay for DNRCC patients were significantly higher than those for non-DNR patients. By contrast, only the daily cost of hospital stay for DNRCC-Arrest patients was significantly higher than for non-DNR patients. The comparisons between the 88 DNRCC and 2,051 non-DNR patients, and between the 188 DNRCC-Arrest and 2,051 non-DNR patients are shown in Table 1 (also see Additional file 1: Table S1; see Additional file 2: Table S2.

**Table 1:** **Comparison of patient characteristics between DNRCC and non-DNR patients, and between DNRCC-Arrest and non-DNR patients before matching** ^**a,b**^

	**DNRCC (n = 88)**	***P*** **value** ^**c**^	**Non-DNR (n = 2,051)**	***P*** **value** ^**d**^	**DNRCC-Arrest (n = 188)**
Age	63.61 ± 15.52	<0.01	54.23 ± 17.76	<0.01	66.83 ± 16.28
Sex (male)	52 (59.09%)	0.19	1065 (51.93%)	0.82	96 (51.06%)
APACHE II minus GCS	23.60 ± 8.05	<0.01	15.20 ± 6.83	<0.01	21.39 ± 7.27
GCS	7.68 ± 4.60	<0.01	12.07 ± 4.00	<0.01	10.57 ± 4.39
Length of stay in the ICU by hour	91.99 ± 130.42	0.16	72.00 ± 86.21	<0.01	120.04 ± 130.75
Length of stay in the hospital by hour	155.47 ± 170.19	0.03	197.58 ± 205.09	<0.01	256.77 ± 210.98
Admission delay^e^	34 (38.64%)	<0.01	463 (22.57%)	0.03	56 (29.79%)
Intubated during ICU stay	56 (63.64%)	<0.01	552 (26.91%)	<0.01	73 (38.83%)
Prior end-of-life decision documented	6 (6.82%)	<0.01	43 (2.10%)	0.15	7 (3.72%)
Cared for by only one intensivist^f^	51 (57.95%)	0.36	1288 (62.80%)	<0.01	86 (45.74%)
Elixhauser comorbidity measures^g^		<0.01 ~ 0.97		<0.01 ~ 0.91	
Insurance type		<0.01		<0.01	
Private	41 (46.59%)		611 (29.79%)		74 (39.36%)
Medicare only	11 (12.50%)		257 (12.53%)		38 (20.21%)
Medicaid only	14 (15.91%)		506 (24.67%)		34 (18.09%)
Medicare and Medicaid	17 (19.32%)		316 (15.41%)		29 (15.43%)
None	5 (5.68%)		361 (17.60%)		13 (6.91%)
Source of admission to the ICU		0.02		0.10	
Emergency department	53 (60.23%)		1513 (73.77%)		124 (65.96%)
Floor^h^	31 (35.23%)		413 (20.14%)		53 (28.19%)
Other ICU	2 (2.27%)		49 (2.39%)		6 (3.19%)
Outside hospital	1 (1.14%)		26 (1.27%)		2 (1.06%)
Miscellaneous/others	1 (1.14%)		50 (2.44%)		3 (1.60%)
Race/ethnicity		0.47		<0.01	
White American	60 (68.18%)		1277 (62.26%)		141 (75%)
African American	23 (26.14%)		602 (29.35%)		37 (19.68%)
Other	5 (5.68%)		172 (8.39%)		10 (5.32%)
ICU admission diagnosis^i^		<0.01		<0.01	
Medical–respiratory diseases	37 (42.53%)		587 (28.96%)		74 (39.36%)
Medical–gastrointestinal diseases	6 (6.9%)		321 (15.84%)		23 (12.23%)
Medical–cardiovascular diseases	18 (20.69%)		347 (17.12%)		50 (26.60%)
Medical–neurological diseases	20 (22.99%)		334 (16.48%)		35 (18.62%)
Other	6 (6.9%)		438 (21.61%)		6 (3.19%)
Daily cost of ICU stay	7592 ± 7465	0.02	5691 ± 4578	0.16	5302 ± 3546
Daily cost of hospital stay	5966 ± 7374	<0.01	3191 ± 2033	<0.01	3878 ± 3452
Daily discretionary cost of ICU stay	2666 ± 3239	0.03	1878 ± 2416	0.77	1822 ± 2466

APACHE, Acute Physiology and Chronic Health Evaluation; DNR, Do-Not-Resuscitate; DNRCC, Do-Not-Resuscitate Comfort Care; DNRCC-Arrest Do-Not-Resuscitate Comfort Care-Arrest; GCS, Glasgow Coma Scale; ICU, medical intensive care unit.

^a^The statistical association between two categorical variables was examined using the χ^2^ test.

^b^The statistical association between a categorical variable and a continuous variable was examined using Student’s *t*-test.

^c^Statistical significance for the comparisons between DNRCC and non-DNR patients.

^d^Statistical significance for the comparisons between DNRCC-Arrest and non-DNR patients.

^e^“Admission delay” means that the time between hospital admission and ICU admission was not zero.

^f^“Cared for by only one intensivist” means that the patient was cared for by only one intensivist during their ICU stay.

^g^See Additional file 2: Table S2 for the comparison of Elixhauser comorbidity measures between DNRCC and non-DNR, and between DNRCC-Arrest and non-DNR before propensity score matching.

^h^“Floor” means that the patient was admitted to other departments before admitting to ICU.

^i^“ICU admission diagnosis” for non-DNR patients had 24 missing data items.

### DNRCC and non-DNR patients

Model 1 included 40 confounding variables. A total of 88 matched pairs were generated. Propensity scores of the 88 DNRCC patients ranged from 0.0007 to 0.8583, with a mean ± SD of 0.2821 ± 0.2278. Propensity scores of the 88 non-DNR patients ranged from 0.0007 to 0.7627, with a mean ± SD of 0.2454 ± 0.1748. The mean ± SD of the propensity score differences of the 88 matched pairs was 0.0497 ± 0.1140. For the 88 matched pairs, none of the 40 confounding variables was significantly different between the two groups (Table 2; see Additional file 3: Table S3).

**Table 2:** **Comparison of patient characteristics and medical care between DNRCC and non-DNR patients after matching** ^**a,b**^

	**DNRCC (n = 88)**	**Non-DNR (n = 88)**	***P*** **value**	**SD**
Patient characteristics				
Age	63.61 ± 15.52	64.01 ± 16.98	0.87	−0.02
Sex (male)	52 (59.09%)	54 (61.36%)	0.76	−0.05
APACHE II minus GCS	23.60 ± 8.05	23.31 ± 7.41	0.80	0.04
GCS	7.68 ± 4.60	7.61 ± 4.33	0.92	0.02
Length of stay in the ICU by hour	91.99 ± 130.42	107.74 ± 132.81	0.43	−0.12
Length of stay in the hospital by hour	155.47 ± 170.19	184.31 ± 161.66	0.25	−0.17
Admission delay^c^	34 (38.64%)	29 (32.95%)	0.43	0.12
Intubated during ICU stay	56 (63.64%)	56 (63.64%)	1.00	0
Prior end-of-life decision documented	6 (6.82%)	7 (7.95%)	0.77	−0.04
Cared for by only one intensivist^d^	51 (57.95%)	52 (59.09%)	0.88	−0.02
Elixhauser comorbidity measures^e^	–	–	0.15 ~ 1.00	−0.14 ~ 0.22
Insurance type			0.78	
Private	41 (46.59%)	41 (46.59%)		0
Medicare only	11 (12.50%)	7 (7.95%)		0.15
Medicaid only	14 (15.91%)	19 (21.59%)		0.15
Medicare and Medicaid	17 (19.32%)	17 (19.32%)		0
None	5 (5.68%)	4 (4.55%)		0.05
Source of admission to ICU			0.88	
Emergency department	53 (60.23%)	58 (65.91%)		−0.12
Floor^f^	31 (35.23%)	25 (28.41%)		0.15
Other ICU	2 (2.27%)	2 (2.27%)		0
Outside hospital	1 (1.14%)	1 (1.14%)		0
Miscellaneous/other	1 (1.14%)	2 (2.27%)		−0.09
Race/ethnicity			0.99	
White American	60 (68.18%)	61 (69.32%)		−0.02
African American	23 (26.14%)	22 (25%)		0.03
Other	5 (5.68%)	5 (5.68%)		0
ICU admission diagnosis^g^			0.97	
Medical–respiratory diseases	37 (42.53%)	35 (40.70%)		0.05
Medical–gastrointestinal diseases	6 (6.9%)	5 (5.81%)		−0.10
Medical–cardiovascular diseases	18 (20.69%)	16 (18.60%)		0.06
Medical–neurological diseases	20 (22.99%)	23 (26.74%)		−0.08
Others	6 (6.9%)	7 (8.14%)		−0.05
Medical care				
Daily cost of ICU stay	7592 ± 7465	6528 ± 4829	0.26	
Daily cost of hospital stay	5966 ± 7374	4616 ± 3989	0.13	
Daily discretionary cost of ICU stay	2666 ± 3239	2286 ± 2371	0.38	
Pharmacological/electrical cardioversion	15 (17.05%)	20 (22.73%)	0.35	
Vasopressor	36 (40.91%)	23 (26.14%)	0.04	
Intravenous antibiotics	66 (75%)	71 (80.68%)	0.36	
Renal replacement therapy	4 (4.55%)	18 (20.45%)	<0.01	
Blood component transfusion	25 (28.41%)	26 (29.55%)	0.87	
Central venous line placement	36 (40.91%)	45 (51.14%)	0.17	
Pain control using morphine/fentanyl	75 (85.23%)	42 (47.73%)	<0.01	
Pastoral care	42 (47.73%)	30 (34.09%)	0.07	
Hospice/palliative care consultation	27 (30.68%)	8 (9.09%)	<0.01	

APACHE, Acute Physiology and Chronic Health Evaluation; DNR, Do-Not-Resuscitate; DNRCC, Do-Not-Resuscitate Comfort Care; DNRCC-Arrest Do-Not-Resuscitate Comfort Care-Arrest; GCS, Glasgow Coma Scale; ICU, medical intensive care unit.

^a^The statistical association between two categorical variables was examined using the χ^2^ test.

^b^The statistical association between a categorical variable and a continuous variable was examined using Student’s *t*-test.

^c^“Admission delay” means that the time between hospital admission and ICU admission was not zero.

^d^“Cared for by only one intensivist” means that the patient was cared for by only one intensivist during their ICU stay.

^e^See Additional file 3: Table S3 for the comparison of Elixhauser comorbidity measures between DNRCC and non-DNR after propensity score matching.

^f^“Floor” means that the patient was admitted to other departments before admitting to ICU.

^g^“ICU admission diagnosis” was missing for two non-DNR patients and one DNRCC patient.

Although the daily cost of ICU stay, daily cost of hospital stay, and daily discretionary cost of ICU stay were higher for DNRCC patients than for non-DNR patients, the differences did not have statistical significance (*P* = 0.13 to 0.38). Four aggressive interventions (pharmacological/electrical cardioversion, intravenous antibiotics, blood component transfusion, and central venous line placement) were not significantly different between DNRCC and non-DNR patients. Non-DNR patients received more renal replacement therapy (*P* < 0.01) than DNRCC patients, while DNRCC patients significantly received more vasopressor (*P* = 0.04), pain control using morphine/fentanyl (*P* < 0.01), and hospice/palliative care consultation (*P* < 0.01) than non-DNR patients (Table 2).

We found that all six aggressive interventions significantly decreased, and all three comfort care measures significantly increased after the order was in effect (Table 3).

**Table 3:** **Comparison of six aggressive interventions and three comfort care measures provided to the 88 DNRCC patients before and after the order was in effect** ^**a**^

	**Before DNRCC was in effect (n = 88)**		**After DNRCC was in effect (n = 88)**		***P*** **value**
	**Provided**	**Not provided**	**Provided**	**Not provided**	
Aggressive intervention					
Pharmacological/electrical cardioversion	14 (15.9%)	74 (84.1%)	2 (2.27%)	86 (97.73%)	<0.01
Vasopressor	36 (40.9%)	52 (59.1%)	0 (0%)	88 (100%)	<0.01
Intravenous antibiotics	66 (75%)	22 (25%)	8 (9.09%)	80 (90.91%)	<0.01
Renal replacement therapy	4 (4.54%)	84 (95.46%)	0 (0%)	88 (100%)	0.05
Blood component transfusion	25 (28.4%)	63 (71.6%)	1 (1.13%)	87 (98.87%)	<0.01
Central venous line placement	36 (40.9%)	52 (59.1%)	6 (6.81%)	82 (93.19%)	<0.01
Comfort care measure					
Pain control using morphine/fentanyl	35 (39.77%)	53 (60.23%)	75 (85.22%)	13 (14.78%)	<0.01
Pastoral care	22 (25%)	66 (75%)	34 (38.63%)	54 (61.37%)	0.02
Hospice/palliative care consultation	5 (5.68%)	83 (94.32%)	24 (27.27%)	64 (72.73%)	<0.01

DNR, Do-Not-Resuscitate; DNRCC, Do-Not-Resuscitate Comfort Care.

^a^The association between before DNRCC and after DNRCC for each intervention was examined using McNemar’s test.

### DNRCC-Arrest and non-DNR patients

Model 2 including 42 confounding variables was built. There were 188 matched pairs. Propensity scores of the 188 DNRCC-Arrest patients ranged from 0.0076 to 0.8182, with a mean ± SD of 0.2257 ± 0.1565. Propensity scores of the 188 non-DNR patients ranged from 0.0076 to 0.8264, with a mean ± SD of 0.2256 ± 0.1558. The mean ± SD of the propensity score differences of the 188 matched pairs was 0.0055 ± 0.0305.

For the 188 matched pairs, none of the 42 confounding variables was significantly different between the DNRCC-Arrest and non-DNR patients (*P* = 0.13 to 1.00) (Table 4; see Additional file 4: Table S4). Although the daily cost of ICU stay and the daily discretionary cost of ICU stay were a little higher in non-DNR patients, and the daily cost of hospital stay was a little higher in DNRCC-Arrest patients, those differences did not have statistical significance (*P* = 0.14 to 0.43). Medical care provided to patients as indicated by the six aggressive interventions was not significantly different between DNRCC-Arrest and non-DNR patients (*P* = 0.18 to 1.00). The three comfort care measures were not significantly different between DNRCC-Arrest and non-DNR patients (*P* = 0.25 to 0.55).

**Table 4:** **The comparison of patient characteristics and medical care between DNRCC-Arrest and non-DNR patients after matching** ^**a,b**^

	**DNRCC-Arrest (n = 188)**	**Non-DNR (n = 188)**	***P*** **value**	**SD**
Patient characteristics				
Age	66.83 ± 16.28	66.70 ± 14.92	0.93	0.01
Sex (male)	96 (51.06%)	97 (51.60%)	0.92	−0.01
APACHE II minus GCS	21.39 ± 7.27	21.24 ± 6.61	0.79	0.02
GCS	10.57 ± 4.39	10.91 ± 4.30	0.45	−0.08
Length of stay in the ICU by hour	120.04 ± 130.75	119.94 ± 151.84	0.99	0.001
Length of stay in the hospital by hour	256.77 ± 210.98	254.32 ± 225.38	0.91	0.01
Admission delay^c^	56 (29.79%)	59 (31.38%)	0.74	−0.04
Intubated during ICU stay	73 (38.83%)	67 (35.64%)	0.52	0.07
Prior end-of-life decision documented	7 (3.72%)	8 (4.26%)	0.79	−0.03
Cared for by only one intensivist^d^	86 (45.74%)	89 (47.34%)	0.76	−0.03
Elixhauser comorbidity measures^e^			0.13 ~ 1.00	−0.14 ~ 0.16
Insurance type			0.90	
Private	74 (39.36%)	70 (37.23%)		0.04
Medicare only	38 (20.21%)	41 (21.81%)		−0.04
Medicaid only	34 (18.09%)	38 (20.21%)		−0.05
Medicare and Medicaid	29 (15.43%)	24 (12.77%)		0.08
None	13 (6.91%)	15 (7.98%)		−0.04
Source of admission to ICU			0.85	
Emergency department	124 (65.96%)	118 (62.77%)		0.07
Floor^f^	53 (28.19%)	54 (28.72%)		−0.01
Other ICU	6 (3.19%)	10 (5.32%)		−0.11
Outside hospital	2 (1.06%)	3 (1.60%)		−0.05
Miscellaneous	3 (1.60%)	3 (1.60%)		0
Race/ethnicity			0.81	
White American	141 (75%)	137 (72.87%)		0.07
African American	37 (19.68%)	42 (22.34%)		−0.01
Other	10 (5.32%)	9 (4.79%)		−0.11
ICU admission diagnosis			0.98	
Medical–respiratory diseases	74 (39.36%)	77 (40.96%)		0.07
Medical–gastrointestinal diseases	23 (12.23%)	21 (11.17%)		−0.01
Medical–cardiovascular diseases	50 (26.60%)	53 (28.19%)		−0.11
Medical–neurological diseases	35 (18.62%)	32 (17.02%)		−0.05
Other	6 (3.19%)	5 (2.66%)		0
Medical care				
Daily cost of ICU stay	5302 ± 3546	5915 ± 4358	0.14	
Daily cost of hospital stay	3878 ± 3452	3605 ± 2505	0.38	
Daily discretionary cost of ICU stay	1822 ± 2466	2018 ± 2312	0.43	
Pharmacological/electrical cardioversion	42 (22.34%)	40 (21.28%)	0.80	
Vasopressor	44 (23.40%)	44 (23.40%)	1.00	
Intravenous antibiotics	138 (73.40%)	149 (79.26%)	0.18	
Renal replacement therapy	16 (8.51%)	16 (8.51%)	1.00	
Blood component transfusion	61 (32.45%)	68 (36.17%)	0.45	
Central venous line	65 (34.57%)	74 (39.36%)	0.34	
Pain control using morphine/fentanyl	68 (36.17%)	79 (42.02%)	0.25	
Pastoral care	54 (28.72%)	61 (32.45%)	0.43	
Hospice/palliative care consultation	15 (7.98%)	12 (6.38%)	0.55	

APACHE, Acute Physiology and Chronic Health Evaluation; DNR Do-Not-Resuscitate; DNRCC, Do-Not-Resuscitate Comfort Care; DNRCC-Arrest Do-Not-Resuscitate Comfort Care-Arrest; GCS, Glasgow Coma Scale; ICU, medical intensive care unit.

^a^The statistical association between two categorical variables was examined using the χ^2^ test.

^b^The statistical association between a categorical variable and a continuous variable was examined using Student’s *t*-test.

^c^“Admission delay” means that the time between hospital admission and ICU admission was not zero.

^d^“Cared for by only one intensivist” means that the patient was cared for by only one intensivist during their ICU stay.

^e^See Additional file 4: Table S4 for the comparison of Elixhauser comorbidity measures between DNRCC-Arrest and non-DNR after propensity score matching.

^f^“Floor” means that the patient was admitted to other departments before admitting to ICU.

We found that none of the aggressive interventions significantly decreased after the orders were written, and all three comfort care measures significantly increased after the order was in effect (*P* < 0.01) (Table 5).

**Table 5:** **The comparison of six aggressive interventions and three comfort care measures provided to the 188 DNRCC-Arrest patients before and after the order came into effect** ^**a**^

	**Before DNRCC-Arrest came into effect (n = 188)**		**After DNRCC-Arrest came into effect (n = 188)**		***P*** **value**
	**Provided**	**Not provided**	**Provided**	**Not provided**	
Aggressive intervention					
Pharmacological/electrical cardioversion	36 (19.14%)	152 (80.86%)	39 (20.74%)	149 (79.26%)	0.32
Vasopressor	33 (17.55%)	155 (82.45%)	34 (18.08%)	154 (81.92%)	0.83
Intravenous antibiotics	122 (64.89%)	66 (35.11%)	133 (70.74%)	55 (29.26%)	0.02
Renal replacement therapy	12 (6.38%)	176 (93.62%)	12 (6.38%)	176 (93.62%)	1.00
Blood component transfusion	42 (22.34%)	146 (77.66%)	43 (22.87%)	145 (77.13%)	0.87
Central venous line placement	59 (31.38%)	129 (68.62%)	64 (34.04%)	124 (65.96%)	0.06
Comfort care measure					
Pain control using morphine/fentanyl	44 (23.4%)	144 (76.6%)	62 (32.97%)	126 (67.03%)	<0.01
Pastoral care	13 (6.91%)	175 (93.09%)	46 (24.46%)	142 (75.54%)	<0.01
Hospice/palliative care consultation	1 (0.53%)	187 (99.47%)	15 (7.97%)	173 (92.03%)	<0.01

DNR, Do-Not-Resuscitate; DNRCC, Do-Not-Resuscitate Comfort Care.

^a^The association between before DNRCC-Arrest and after DNRCC-Arrest for each intervention was examined using McNemar’s test.

### Sensitivity analysis

Age was excluded from the propensity score model for DNRCC and non-DNR patients. A total of 39 confounding variables were included. Only one of the confounding variables (alcohol/drug abuse; *P* = 0.03), was not balanced (see Additional file 5: Table S5). We then conducted multivariate linear regression of the daily cost of ICU stay, the daily cost of hospital stay, the daily discretionary cost of ICU stay on alcohol/drug abuse, propensity score, and DNR decisions (see Additional file 6: Table S6). After controlling for the propensity score and alcohol/drug abuse, we found that the daily cost of ICU stay (*P* = 0.21), the daily cost of hospital stay (*P* = 0.09), and the daily discretionary cost of ICU stay (*P* = 0.33) were not significantly different between DNRCC and non-DNR patients.

A total of 41 confounding variables, excluding age, were included in the propensity score model for DNRCC-Arrest and non-DNR. All of the confounding variables were balanced (see Additional file 7: Table S7). We then conducted Student’s *t*-test to examine the associations between the daily cost of ICU stay, the daily cost of hospital stay, the daily discretionary cost of ICU stay and DNR decisions. After controlling for the confounding variables, we found that the daily cost of ICU stay (*P* = 0.11), the daily cost of hospital stay (*P* = 0.56), and the daily discretionary cost of ICU stay (*P* = 0.08) were not significantly different between DNRCC-Arrest and non-DNR patients.

Our sensitivity analyses for DNRCC and non-DNR and for DNRCC-Arrest and non-DNR showed no significant difference for the three costs between DNRCC and non-DNR, and between DNRCC-Arrest and non-DNR, which were similar to the results derived from Model 1 (Table 2) and Model 2 (Table 4).

## Discussion

### Main findings

This study examined the medical care provided to DNRCC and DNRCC-Arrest patients in a medical ICU, as indicated by the three medical costs, six aggressive interventions, and three comfort care measures. We found that DNRCC patients received significantly fewer aggressive interventions and more comfort care after the order was initiated. By contrast, most of the six aggressive interventions provided to DNRCC-Arrest patients were not significantly different before and after the order was initiated. The three comfort care measures provided to DNRCC-Arrest patients significantly increased after the order was initiated. In addition, the daily cost of ICU stay, the daily cost of hospital stay, and the daily discretionary cost of ICU stay were not significantly different between DNRCC and non-DNR patients, or between DNRCC-Arrest and non-DNR patients. According to our study, when medical care provided to DNR patients is clearly indicated, as in Ohio’s Do-Not-Resuscitate law, healthcare professionals will provide the medical care determined by patient/surrogate decision-makers and healthcare professionals rather than blindly decreasing medical care.

Several studies have focused on the attitudes of physicians and nurses toward the medical care provided to patients. Henneman *et al*. reported that nurses would be significantly less likely to perform a variety of physiologic monitoring techniques and interventions for DNR than for non-DNR patients. The authors raised concerns that DNR may be misinterpreted as more than “no CPR in face of cardiac or respiratory arrest” [20]. Beach *et al*. also examined the effect of DNR orders on physicians’ decisions to provide medical care. They found that physicians were significantly less likely to provide aggressive interventions such as blood culture and central venous line placement to DNR than non-DNR patients [21]. Park *et al*. also concluded that nurses were less likely to provide care such as simple massage, reporting the patient’s condition, and central venous pressure monitoring to DNR patients [5]. All these studies, using hypothetical case scenarios, showed that DNR patients tend to receive less medical care than non-DNR patients.

Some studies further investigated the influence of DNR orders on the relationship between medical care and DNR orders using real cases. Jackson *et al*. found that, in the Worcester Heart Attack Study, hospitalized patients with acute myocardial infarction were significantly less likely to be treated with effective cardiac medications such as aspirin, β-blockers, thrombolytics, and cardiac catheterization if they had DNR orders written [7]. Chen *et al*. showed that DNR patients with acute heart failure were less likely than non-DNR patients to have their left ventricular function assessed, or to receive anticoagulation, angiotensin-converting-enzyme inhibitors, angiotensin receptor blockers, or non-pharmacologic counseling [6]. Baker *et al*. reported that the most frequently discontinued medical interventions for pediatric oncologic patients were blood drawn for laboratory check, chemotherapy, antibiotics, and parenteral nutrition [22].

In the current study, we examined the level of medical care patients received after one of two specific DNR protocols was put into place. We expected that DNRCC patients would receive fewer aggressive interventions than non-DNR patients. However, the results showed that the aggressive interventions provided to DNRCC patients were not significantly different from those provided to non-DNR patients. This is because the comparison of aggressive interventions between DNRCC and non-DNR patients using propensity score matching could not distinguish between aggressive interventions provided to the patients before the initiation of DNRCC, and those provided to the patients after the initiation of DNRCC.

To directly measure the influence of DNRCC/DNRCC-Arrest on the medical care provided to patients, we conducted further analysis using one group before-and-after design. We found that DNRCC patients received fewer aggressive interventions and more comfort care measures after the orders were written. In comparison, none of the six aggressive interventions and three comfort care measures significantly decreased after the DNRCC-Arrest orders were written.

DNRCC patients will receive only comfort care after the DNRCC order is in effect, which is similar to the philosophy of palliative care services. Providing comfort/palliative care to patients admitted to ICU has the potential to enhance the quality of care by alleviating pain, dyspnea, abnd thirst, and shortening the length of stay in ICU while not changing patient mortality or satisfaction [23-25]. If a patient had a DNRCC order written during an ICU stay, they the following measures occurred: 1) life-extending aggressive interventions were gradually withdrawn; (2) comfort care measures, such as hospice/palliative care consultation, the use of morphine and so on were gradually added, based on discussions between healthcare professionals and patients/family members; and 3) there was potential transfer of the patient to another non-ICU bed for further care.

Our study demonstrated that healthcare professionals provided the medical care to DNR patients in accordance with Ohio’s Do-Not-Resuscitate law. Our study results further support the American Medical Association statement in 1991 that DNR precludes only CPR, and should not preclude medical care provided to patients prior to the initiation of CPR [2].

### Strengths and limitations

Our study makes two important contributions to the literature about DNR patients. This is the first study, to our knowledge, to examine the medical care provided to DNR patients under two distinct DNR protocols. The protocols resulted in two different patterns of care, consistent with the intent of the Ohio’s Do-Not-Resuscitate law. Second, our study estimated the actual medical care provided to DNR patients, in contrast to previous studies that used hypothetical cases.

The study has several limitations. The first concern is about its generalizability. It was conducted at a single medical center. However, we believe that the results may be generalizable as much as a multi-center study because: 1) when we compared our dataset with other multi-center studies using the indicators of age, severity of illness, and race/ethnicity, we found that our dataset showed similar findings to those of several multi-center studies; 2) the age and sex distributions of study population in the vicinity of our study site (Cuyahoga County in the State of Ohio) are similar to those of the USA overall. According to the US census of 2010, 49.2% of the population of the USA were males, and the median age was 37.2 years; the figures for Cuyahoga County were 47.4% and 40.2 years, respectively [26]. In addition, the propensity score methodology may also reduce the generalizability of the study results. The objective of this study was to examine the medical care provided to DNRCC, DNRCC-Arrest, or non-DNR patients. For comparing like with like, we used propensity score methodology to compare the DNRCC-Arrest/DNRCC patients with a subset of non-DNR patients who were similar in all measurable ways, except that they did not have a DNRCC-Arrest/DNRCC decision [15,27]. The subset of non-DNR patients selected using propensity score methodology was not a good representative of the non-DNR patients as a whole. Therefore, the study results are favorable for being generalizable to non-DNR patients who are similar to DNRCC-Arrest/DNRCC patients, but not to the non-DNR patient subset who are different from DNRCC-Arrest/DNRCC patients.

The second limitation is that ours was a retrospective and observational cohort study, not a prospective and randomized trial. Although we tried to adjust for all available confounding variables using propensity scores, there may have been other potential confounders. However, assigning DNR status to a patient in a randomized trial is not ethically possible.

Third, we estimated medical care provided to DNR and non-DNR patients during the medical ICU stay using the three medical costs, six aggressive interventions, and three comfort care measures. Our study results might not be generalizable to medical care beyond these measures.

The fourth limitation was that our retrospective study was unable to document the discussions, goals, and exact wishes of DNR patients and surrogates. Although the two patterns of care we identified support the intent of the law to allow “customized” DNR care, we can only infer that they are also accurate expressions of patient and surrogate preferences. Prospective studies should further clarify this important issue.

## Conclusions

Some hospitals in the USA already have an institutional policy regarding the two protocols of DNR orders similar to Ohio’s Do-Not-Resuscitate law [28]. Our study is the first to examine medical care provided to DNRCC and DNRCC-Arrest patients in the State of Ohio, where a Do-Not-Resuscitate law has been in effect for some time. Our results suggest DNR protocols that require consideration and documentation of wider treatment intent may help alleviate concern that DNR patients will receive either too much or too little medical care. Whether through specific institutional DNR policies or state law, the simple and vague DNR order should become a thing of the past. Interpretation of DNR should not be left to the imagination.

## Additional files

Additional file 1: Table S1. The median and percentiles of each continuous variable for DNRCC, non-DNR and DNRCC-Arrest patients. Additional file 2: Table S2. The comparison of Elixhauser comorbidity measures between DNRCC and non-DNR, and between DNRCC-Arrest and non-DNR before propensity score matching. Additional file 3: Table S3. The comparison of Elixhauser comorbidity measures between DNRCC and non-DNR patients after propensity score matching. Additional file 4: Table S4. The comparison of Elixhauser comorbidity measures between DNRCC-Arrest and non-DNR patients after propensity score matching. Additional file 5: Table S5. The comparison of patient characteristics and medical care between DNRCC and non-DNR patients after matching using propensity score model excluding age. Additional file 6: Table S6. Multivariate linear regression models on the daily cost of ICU stay, daily cost of hospital stay, daily discretionary cost of ICU stay for DNRCC and non-DNR patients after matching using propensity score model excluding age. Additional file 7: Table S7. The comparison of patient characteristics and medical care between DNRCC-Arrest and non-DNR patients after matching using propensity score model excluding age.

## Abbreviations

APACHE: Acute Physiology and Chronic Health Evaluation
DNR: Do-not-resuscitate
DNRCC: Do-Not-Resuscitate Comfort Care
DNRCC-Arrest: Do-Not-Resuscitate Comfort Care-Arrest
GCS: Glasgow Coma Score
ICU: Intensive care unit
