# Supplementary material for: Two distinct Do-Not-Resuscitate protocols leaving less to the imagination: an observational study using propensity score matching
Source: BMC Med. 2014 Aug 29;12:146. doi: 10.1186/s12916-014-0146-x (PMC4156651; doi:10.1186/s12916-014-0146-x)
Supplement: Additional file 1: Table S1. — The median and percentiles of each continuous variable for DNRCC, non-DNR and DNRCC-Arrest patients. [file 12916_2014_146_MOESM1_ESM.docx]

**Supplementary Table 1. The median and percentiles of each continuous variable for DNRCC, Non-DNR and DNRCC-Arrest patients.**

|  | **DNRCC, N = 88**  **Median (25^th^~75^th^ percentile)** | **Non-DNR, N = 2,051**  **Median (25^th^~75^th^ percentile)** | **DNRCC-Arrest, N = 188**  **Median (25^th^~75^th^ percentile)** |
| --- | --- | --- | --- |
| **Age** | 63 (52~77) | 53 (42~68) | 69 (55~79.75) |
| **APACHE II minus GCS** | 22.5 (17~29) | 15 (10~20) | 21 (17~26) |
| **GCS** | 7 (3~13) | 14 (10~15) | 12 (7~15) |
| **Length of stay in the ICU by hour** | 42.33 (23.43~97.53) | 45.25 (27.8~77.25) | 75.72 (43.49~147.61) |
| **Length of stay in the hospital by hour** | 96.49 (36.98~219.33) | 131.17 (74.15~246.47) | 213.52 (116.36~341.68) |
| **Daily cost of ICU stay** | 9301 (5953~20886) | 4574 (3861~6011) | 4343 (3755~5452) |
| **Daily cost of hospital stay** | 13468 (7344~30458) | 2752 (2165~3551) | 2989 (2230~3787) |
| **Daily discretionary cost of ICU stay** | 2778 (1433~6458) | 1250 (830~2021) | 1212 (813~1918) |

Abbreviation List: APACHE II = Acute Physiology and Chronic Health Evaluation II; GCS = Glasgow Coma Scale; DNRCC = Do-not-resuscitate Comfort Care; DNRCC-Arrest = Do-not-resuscitate Comfort Care Arrest; DNR = Do-not-resuscitate; ICU = medical intensive care unit.
