# Supplementary material for: Two distinct Do-Not-Resuscitate protocols leaving less to the imagination: an observational study using propensity score matching
Source: BMC Med. 2014 Aug 29;12:146. doi: 10.1186/s12916-014-0146-x (PMC4156651; doi:10.1186/s12916-014-0146-x)
Supplement: Additional file 2: Table S2. — The comparison of Elixhauser comorbidity measures between DNRCC and non-DNR, and between DNRCC-Arrest and non-DNR before propensity score matching. [file 12916_2014_146_MOESM2_ESM.docx]

**Supplementary Table 2. The comparison of Elixhauser comorbidity measures between DNRCC and Non-DNR, and between DNRCC-Arrest and Non-DNR before propensity score matching.**

|  | **DNRCC**  **N = 88** | ***p* value** *^a^* | **Non-DNR**  **N = 2,051** | | ***p* value** *^b^* | **DNRCC-Arrest**  **N = 188** |
| --- | --- | --- | --- | --- | --- | --- |
| **Congestive heart failure** | 18 (20.45%) | 0.57 | 370 (18.04%) | | 0.28 | 49 (26.06%) |
| **Cardiac arrhythmias** | 17 (19.32%) | 0.27 | 307 (14.97%) | | <0.01 | 43 (22.87%) |
| **Valvular disease** | 3 (3.41%) | 0.54 | 99 (4.83%) | | 0.53 | 11 (5.85%) |
| **Pulmonary circulatory disorders** | 2 (2.27%) | 0.67 | 63 (3.07%) | | 0.38 | 8 (4.26%) |
| **Peripheral vascular disorders** | 7 (7.95%) | 0.56 | 131 (6.39%) | | 0.05 | 19 (10.11%) |
| **Hypertension** | 33 (37.5%) | 0.57 | 831 (40.52%) | | 0.46 | 71 (37.77%) |
| **Paralysis** | 6 (6.82%) | 0.97 | 138 (6.73%) | | <0.01 | 23 (12.23%) |
| **Other neurological disorders** | 12 (13.64%) | 0.01 | 134 (6.53%) | | <0.01 | 23 (12.23%) |
| **Chronic pulmonary disease** | 21 (23.86%) | 0.32 | 589 (28.72%) | | 0.28 | 61 (32.45%) |
| **Diabetes, umcomplicated** | 24 (27.27%) | 0.57 | 505 (24.62%) | | 0.55 | 50 (26.6%) |
| **Diabetes, complicated** | 3 (3.41%) | 0.44 | 108 (5.27%) | | 0.78 | 9 (4.79%) |
| **Hypothyroidism** | 10 (11.36%) | 0.22 | 159 (7.75%) | | 0.03 | 23 (12.23%) |
| **Renal failure** | 15 (17.05%) | 0.17 | 249 (12.14%) | | 0.38 | 27 (14.36%) |
| **Liver disease** | 15 (17.05%) | 0.11 | 235 (11.46%) | | 0.33 | 26 (13.83%) |
| **Peptic ulcer disease excluding bleeding** | 2 (2.27%) | 0.67 | 63 (3.07%) | | 0.25 | 3 (1.6%) |
| **AIDS** | 6 (6.82%) | 0.02 | 53 (2.58%) | | 0.18 | 8 (4.26%) |
| **Lymphoma** | 3 (3.41%) | 0.16 | 31 (1.51%) | | 0.51 | 4 (2.13%) |
| **Solid tumor without metastasis** | 22 (25%) | <0.01 | 192 (9.36%) | | <0.01 | 38 (20.21%) |
| **Rheumatoid arthritis/collagen vascular diseases** | 8 (9.09%) | 0.67 | 161 (7.85%) | | 0.33 | 11 (5.85%) |
| **Coagulopathy** | 7 (7.95%) | 0.06 | 80 (3.9%) | | <0.01 | 18 (9.57%) |
| **Weight loss** | 3 (3.41%) | 0.64 | 53 (2.58%) | | 0.01 | 11 (5.85%) |
| **Fluid and electrolyte disorders** | 28 (31.82%) | 0.28 | 546 (26.62%) | | <0.01 | 73 (38.83%) |
| **Blood loss anemia** | 9 (10.23%) | 0.06 | 374  (18.24%) | 40 (1.95%) | 0.39 | 2 (1.06%) |
| **Deficiency anemia** |  |  |  | 334 (16.28%) | 0.91 | 30 (15.96%) |
| **Alcohol abuse** | 14 (15.92%) | 0.28 | 423  (20.62%) | 316 (15.41%) | 0.25 | 23 (12.23%) |
| **Drug abuse** |  |  |  | 167 (8.14%) | <0.01 | 5 (2.66%) |
| **Psychoses** | 2 (2.27%) | 0.02 | 209 (10.19%) | | <0.01 | 3 (1.6%) |
| **Depression** | 1 (1.14%) | <0.01 | 227 (11.07%) | | 0.28 | 16 (8.51%) |

Abbreviation List: DNRCC = Do-not-resuscitate Comfort Care; DNRCC-Arrest = Do-not-resuscitate Comfort Care Arrest; DNR = Do-not-resuscitate.

The statistical association between two categorical variables is examined using Chi-squared test.

For comparing DNRCC with Non-DNR, “Blood loss anemia” and “Deficiency anemias” were collapsed to “Anemia.”

For comparing DNRCC with Non-DNR, “Alcohol abuse” and “Drug abuse” were collapsed to “Alcohol/Drug abuse.”

a This *p* value is the statistical significance for the comparisons between DNRCC and Non-DNR patients.

b This *p* value is the statistical significance for the comparisons between DNRCC-Arrest and Non-DNR patients.
