# Supplementary material for: Two distinct Do-Not-Resuscitate protocols leaving less to the imagination: an observational study using propensity score matching
Source: BMC Med. 2014 Aug 29;12:146. doi: 10.1186/s12916-014-0146-x (PMC4156651; doi:10.1186/s12916-014-0146-x)
Supplement: Additional file 3: Table S3. — The comparison of Elixhauser comorbidity measures between DNRCC and non-DNR patients after propensity score matching. [file 12916_2014_146_MOESM3_ESM.docx]

**Supplementary Table 3. The comparison of Elixhauser comorbidity measures between DNRCC and Non-DNR after propensity score matching.**

|  | **DNRCC**  **N = 88** | **Non-DNR**  **N = 88** | ***p* value** | **SD** |
| --- | --- | --- | --- | --- |
| **Congestive heart failure** | 18 (20.45%) | 12 (13.64%) | 0.23 | 0.18 |
| **Cardiac arrhythmias** | 17 (19.32%) | 18 (20.45%) | 0.85 | -0.03 |
| **Valvular disease** | 3 (3.41%) | 1 (1.14%) | 0.31 | 0.15 |
| **Pulmonary circulatory disorders** | 2 (2.27%) | 1 (1.14%) | 0.56 | 0.09 |
| **Peripheral vascular disorders** | 7 (7.95%) | 9 (10.23%) | 0.60 | -0.08 |
| **Hypertension** | 33 (37.5%) | 32 (36.36%) | 0.88 | 0.02 |
| **Paralysis** | 6 (6.82%) | 7 (7.95%) | 0.77 | -0.04 |
| **Other neurological disorders** | 12 (13.64%) | 12 (13.64%) | 1.00 | 0 |
| **Chronic pulmonary disease** | 21 (23.86%) | 23 (26.14%) | 0.73 | -0.05 |
| **Diabetes, umcomplicated** | 24 (27.27%) | 22 (25%) | 0.73 | 0.05 |
| **Diabetes, complicated** | 3 (3.41%) | 1 (1.14%) | 0.31 | 0.15 |
| **Hypothyroidism** | 10 (11.36%) | 13 (14.77%) | 0.50 | -0.10 |
| **Renal failure** | 15 (17.05%) | 20 (22.73%) | 0.35 | -0.14 |
| **Liver disease** | 15 (17.05%) | 20 (22.73%) | 0.35 | -0.14 |
| **Peptic ulcer disease excluding bleeding** | 2 (2.27%) | 2 (2.27%) | 1.00 | 0 |
| **AIDS** | 6 (6.82%) | 2 (2.27%) | 0.15 | 0.22 |
| **Lymphoma** | 3 (3.41%) | 2 (2.27%) | 0.65 | 0.07 |
| **Solid tumor without metastasis** | 22 (25%) | 21 (23.86%) | 0.86 | 0.03 |
| **Rheumatoid arthritis/collagen vascular diseases** | 8 (9.09%) | 9 (10.23%) | 0.80 | -0.04 |
| **Coagulopathy** | 7 (7.95%) | 6 (6.82%) | 0.77 | 0.04 |
| **Weight loss** | 3 (3.41%) | 2 (2.27%) | 0.65 | 0.07 |
| **Fluid and electrolyte disorders** | 28 (31.82%) | 32 (36.36%) | 0.53 | -0.10 |
| **Anemia** | 9 (10.23%) | 9 (10.23%) | 1.00 | 0 |
| **Alcohol/Drug abuse** | 14 (15.92%) | 15 (17.05%) | 0.84 | -0.03 |
| **Psychoses** | 2 (2.27%) | 4 (4.55%) | 0.41 | -0.13 |
| **Depression** | 1 (1.14%) | 1 (1.14%) | 1.00 | 0 |

Abbreviation List: DNRCC = Do-not-resuscitate Comfort Care; DNR = Do-not-resuscitate; SD = standardized difference.

The statistical association between two categorical variables is examined using Chi-squared test.
