# Supplementary material for: Two distinct Do-Not-Resuscitate protocols leaving less to the imagination: an observational study using propensity score matching
Source: BMC Med. 2014 Aug 29;12:146. doi: 10.1186/s12916-014-0146-x (PMC4156651; doi:10.1186/s12916-014-0146-x)
Supplement: Additional file 4: Table S4. — The comparison of Elixhauser comorbidity measures between DNRCC-Arrest and non-DNR patients after propensity score matching. [file 12916_2014_146_MOESM4_ESM.docx]

**Supplementary Table 4. The comparison of Elixhauser comorbidity measures between DNRCC-Arrest and Non-DNR after propensity score matching.**

|  | **DNRCC-Arrest**  **N = 188** | **Non-DNR**  **N = 188** | ***p* value** | **SD** |
| --- | --- | --- | --- | --- |
| **Congestive heart failure** | 49 (26.06%) | 46 (24.47%) | 0.72 | 0.04 |
| **Cardiac arrhythmias** | 43 (22.87%) | 41 (21.81%) | 0.80 | 0.03 |
| **Valvular disease** | 11 (5.85%) | 8 (4.26%) | 0.48 | 0.07 |
| **Pulmonary circulatory disorders** | 8 (4.26%) | 3 (1.6%) | 0.13 | 0.16 |
| **Peripheral vascular disorders** | 19 (10.11%) | 17 (9.04%) | 0.73 | 0.04 |
| **Hypertension** | 71 (37.77%) | 72 (38.3%) | 0.92 | -0.01 |
| **Paralysis** | 23 (12.23%) | 19 (10.11%) | 0.51 | 0.07 |
| **Other neurological disorders** | 23 (12.23%) | 29 (15.43%) | 0.37 | -0.09 |
| **Chronic pulmonary disease** | 61 (32.45%) | 61 (32.45%) | 1.00 | 0 |
| **Diabetes, uncomplicated** | 50 (26.6%) | 53 (28.19%) | 0.73 | -0.04 |
| **Diabetes, complicated** | 9 (4.79%) | 10 (5.32%) | 0.81 | -0.02 |
| **Hypothyroidism** | 23 (12.23%) | 21 (11.17%) | 0.75 | 0.03 |
| **Renal failure** | 27 (14.36%) | 30 (15.96%) | 0.67 | -0.04 |
| **Liver disease** | 26 (13.83%) | 17 (9.04%) | 0.15 | 0.15 |
| **Peptic ulcer disease excluding bleeding** | 3 (1.6%) | 2 (1.06%) | 0.65 | 0.05 |
| **AIDS** | 8 (4.26%) | 6 (3.19%) | 0.59 | 0.06 |
| **Lymphoma** | 4 (2.13%) | 3 (1.6%) | 0.70 | 0.04 |
| **Solid tumor without metastasis** | 38 (20.21%) | 49 (26.06%) | 0.18 | -0.14 |
| **Rheumatoid arthritis/collagen vascular diseases** | 11 (5.85%) | 5 (2.66%) | 0.13 | 0.16 |
| **Coagulopathy** | 18 (9.57%) | 14 (7.45%) | 0.46 | 0.08 |
| **Weight loss** | 11 (5.85%) | 13 (6.91%) | 0.67 | -0.04 |
| **Fluid and electrolyte disorders** | 73 (38.83%) | 70 (37.23%) | 0.75 | 0.03 |
| **Blood loss anemia** | 2 (1.06%) | 1 (0.53%) | 0.56 | 0.06 |
| **Deficiency anemias** | 30 (15.96%) | 31 (16.49%) | 0.89 | -0.01 |
| **Alcohol abuse** | 23 (12.23%) | 23 (12.23%) | 0.75 | 0.03 |
| **Drug abuse** | 5 (2.66%) | 5 (2.66%) | 1.00 | 0 |
| **Psychoses** | 3 (1.6%) | 1 (0.53%) | 0.32 | 0.10 |
| **Depression** | 16 (8.51%) | 18 (9.57%) | 0.72 | -0.04 |

Abbreviation List: DNRCC-Arrest = Do-not-resuscitate Comfort Care Arrest; DNR = Do-not-resuscitate; SD = standardized difference.

The statistical association between two categorical variables is examined using Chi-squared test.
