# Supplementary material for: Two distinct Do-Not-Resuscitate protocols leaving less to the imagination: an observational study using propensity score matching
Source: BMC Med. 2014 Aug 29;12:146. doi: 10.1186/s12916-014-0146-x (PMC4156651; doi:10.1186/s12916-014-0146-x)
Supplement: Additional file 5: Table S5. — The comparison of patient characteristics and medical care between DNRCC and non-DNR patients after matching using propensity score model excluding age. [file 12916_2014_146_MOESM5_ESM.docx]

**Supplementary Table 5. The comparison of patient characteristics and medical care between DNRCC and Non-DNR patients after matching using propensity score model excluding age.**

|  | **DNRCC**  **N = 88** | **Non-DNR**  **N = 88** | ***p* value** |
| --- | --- | --- | --- |
| ***Patient Characteristics*** |  |  |  |
| **Age** | N/A | N/A | N/A |
| **APACHE II minus GCS** | 23.60±8.05 | 23.89±7.15 | 0.80 |
| **GCS** | 7.68±4.60 | 8.03±4.75 | 0.62 |
| **Length of stay in the ICU by hour** | 91.99±130.42 | 120.47±160.30 | 0.20 |
| **Length of stay in the hospital by hour** | 155.47±170.19 | 193.71±173.80 | 0.14 |
| **Admission delay** *^a^* | 34 (38.64%) | 33 (35.70%) | 0.88 |
| **Gender (male)** | 52 (59.09%) | 50 (56.82%) | 0.76 |
| **Intubated during ICU stay** | 56 (63.64%) | 53 (60.23%) | 0.64 |
| **Prior end-of-life decision documented** | 6 (6.82%) | 5 (5.68%) | 0.76 |
| **Cared for by only one intensivist** *^b^* | 51 (57.95%) | 49 (55.68%) | 0.76 |
| **Elixhauser comorbidity measures** |  |  | 0.03~1.00 *^c^* |
| **Insurance type** |  |  | 0.34 |
| Private | 41 (46.59%) | 44 (50%) |  |
| Medicare only | 11 (12.50%) | 4 (4.55%) |  |
| Medicaid only | 14 (15.91%) | 15 (17.05%) |  |
| Medicare and Medicaid | 17 (19.32%) | 22 (25%) |  |
| None | 5 (5.68%) | 3 (3.41%) |  |
| **Source of admission to ICU** |  |  | 0.83 |
| Emergency department | 53 (60.23%) | 52 (59.09%) |  |
| Floor *^d^* | 31 (35.23%) | 28 (31.82%) |  |
| Other ICU | 2 (2.27%) | 4 (4.55%) |  |
| Outside hospital | 1 (1.14%) | 2 (2.27%) |  |
| Miscellaneous/others | 1 (1.14%) | 2 (2.27%) |  |
| **Race/Ethnicity** |  |  | 0.75 |
| American Whites | 60 (68.18%) | 60 (68.18%) |  |
| African Americans | 23 (26.14%) | 25 (28.41%) |  |
| Others | 5 (5.68%) | 3 (3.41%) |  |
| **ICU admission diagnosis** *^e^* |  |  | 0.68 |
| Medical-respiratory diseases | 37 (42.53%) | 43 (48.86%) |  |
| Medical-gastrointestinal diseases | 6 (6.9%) | 3 (3.41%) |  |
| Medical-cardiovascular diseases | 18 (20.69%) | 21 (23.86%) |  |
| Medical-neurological diseases | 20 (22.99%) | 17 (19.32%) |  |
| Others | 6 (6.9%) | 4 (4.55%) |  |
| ***Medical Care*** |  |  |  |
| **Daily cost of ICU stay** | 7592±7465 | 6193±4721 | 0.14 |
| **Daily cost of hospital stay** | 5966±7374 | 4257±3106 | 0.05 |
| **Daily discretionary cost of ICU stay** | 2666±3239 | 2050±2189 | 0.14 |

Abbreviation List: APACHE II = Acute Physiology and Chronic Health Evaluation II; GCS = Glasgow Coma Scale; DNRCC = Do-not-resuscitate Comfort Care; DNR = Do-not-resuscitate; ICU = medical intensive care unit

The statistical association between two categorical variables is examined using Chi-squared test.

The statistical association between a categorical variable and a continuous variable is examined using Student’s t-test.

a “Admission delay” means that the time between hospital admission and ICU admission was not zero.

b “Cared for by only one intensivist” means that the patient was cared for by only one intensivist during his/her ICU stay.

c Only “Alcohol/Drug use” is significantly different between DNRCC and Non-DNR patients with a *p* value of 0.03. Other Elixhauser comorbidity measures are not significantly different between DNRCC and Non-DNR patients.

d “Floor” means that the patient was admitted to other departments before admitting to ICU.

e “ICU admission diagnosis” of one DNRCC patient was missing.
