# Supplementary material for: Two distinct Do-Not-Resuscitate protocols leaving less to the imagination: an observational study using propensity score matching
Source: BMC Med. 2014 Aug 29;12:146. doi: 10.1186/s12916-014-0146-x (PMC4156651; doi:10.1186/s12916-014-0146-x)
Supplement: Additional file 6: Table S6. — Multivariate linear regression models on the daily cost of ICU stay, daily cost of hospital stay, daily discretionary cost of ICU stay for DNRCC and non-DNR patients after matching using propensity score model excluding age. [file 12916_2014_146_MOESM6_ESM.docx]

**Supplementary Table 6. Multivariate linear regression models on the daily cost of ICU stay, the daily cost of hospital stay, the daily discretionary cost of ICU stay for DNRCC and Non-DNR patients after matching using propensity score model excluding age.**

|  | **Outcome variable =**  **the daily cost of ICU stay**  **(N = 176)** | | **Outcome variable =**  **the daily cost of hospital stay**  **(N = 176)** | | **Outcome variable = the daily discretionary cost of ICU stay**  **(N = 176)** | |
| --- | --- | --- | --- | --- | --- | --- |
|  | **Coefficient** | ***p* value** | **Coefficient** | ***p* value** | **Coefficient** | ***p* value** |
| **DNRCC/Non-DNR (DNRCC=1)** | 1150.07 | 0.21 | 1393.05 | 0.09 | 386.13 | 0.33 |
| **Alcohol/Drug abuse (abuse=1)** | -609.31 | 0.68 | 9.98 | 0.99 | 461.81 | 0.47 |
| **Propensity score** | 9002 | <0.01 | 9081.75 | <0.01 | 5266.74 | <0.01 |
| **Adjusted R square** | 0.0821 | | 0.1126 | | 0.1534 | |

Abbreviation List: DNRCC-Arrest = Do-not-resuscitate Comfort Care; DNR = Do-not-resuscitate; ICU = medical intensive care unit.
