# Supplementary material for: Two distinct Do-Not-Resuscitate protocols leaving less to the imagination: an observational study using propensity score matching
Source: BMC Med. 2014 Aug 29;12:146. doi: 10.1186/s12916-014-0146-x (PMC4156651; doi:10.1186/s12916-014-0146-x)
Supplement: Additional file 7: Table S7. — The comparison of patient characteristics and medical care between DNRCC-Arrest and non-DNR patients after matching using propensity score model excluding age. [file 12916_2014_146_MOESM7_ESM.docx]

**Supplementary Table 7. The comparison of patient characteristics and medical care between DNRCC-Arrest and Non-DNR patients after matching using propensity score model excluding age.**

|  | **DNRCC-Arrest**  **N = 188** | **Non-DNR**  **N = 188** | ***p* value** |
| --- | --- | --- | --- |
| ***Patient Characteristics*** |  |  |  |
| **Age** | N/A | N/A | N/A |
| **APACHE II minus GCS** | 21.39±7.27 | 22.20±7.11 | 0.28 |
| **GCS** | 10.57±4.39 | 10.73±4.63 | 0.73 |
| **Length of stay in the ICU by hour** | 120.04±130.75 | 120.61±125.42 | 0.97 |
| **Length of stay in the hospital by hour** | 256.77±210.98 | 268.04±211.00 | 0.60 |
| **Admission delay** *^a^* | 56 (29.79%) | 63 (33.51%) | 0.44 |
| **Gender (male)** | 96 (51.06%) | 101 (53.72%) | 0.61 |
| **Intubated during ICU stay** | 73 (38.83%) | 76 (40.43%) | 0.75 |
| **Prior end-of-life decision documented** | 7 (3.72%) | 7 (3.72%) | 1.00 |
| **Cared for by only one intensivist** *^b^* | 86 (45.74%) | 84 (44.68%) | 0.84 |
| **Elixhauser comorbidity measures** |  |  | 0.12~1.00 |
| **Insurance type** |  |  | 0.90 |
| Private | 74 (39.36%) | 79 (42.02%) |  |
| Medicare only | 38 (20.21%) | 35 (18.62%) |  |
| Medicaid only | 34 (18.09%) | 34 (18.09%) |  |
| Medicare and Medicaid | 29 (15.43%) | 31 (16.49%) |  |
| None | 13 (6.91%) | 9 (4.79%) |  |
| **Source of admission to ICU** |  |  | 0.72 |
| Emergency department | 124 (65.96%) | 116 (61.70%) |  |
| Floor *^c^* | 53 (28.19%) | 61 (32.45%) |  |
| Other ICU | 6 (3.19%) | 7 (3.72%) |  |
| Outside hospital | 2 (1.06%) | 3 (1.60%) |  |
| Miscellaneous | 3 (1.60%) | 1 (0.53%) |  |
| **Race/Ethnicity** |  |  | 0.41 |
| Americans Whites | 141 (75%) | 132 (70.21%) |  |
| African Americans | 37 (19.68%) | 40 (21.28%) |  |
| Others | 10 (5.32%) | 16 (8.51%) |  |
| **ICU admission diagnosis** |  |  | 1.00 |
| Medical-respiratory diseases | 74 (39.36%) | 75 (39.89%) |  |
| Medical-gastrointestinal diseases | 23 (12.23%) | 24 (12.77%) |  |
| Medical-cardiovascular diseases | 50 (26.60%) | 49 (26.06%) |  |
| Medical-neurological diseases | 35 (18.62%) | 33 (17.55%) |  |
| Others | 6 (3.49%) | 7 (3.72%) |  |
| ***Medical Care*** |  |  |  |
| **Daily cost of ICU stay** | 5302±3546 | 5957±4358 | 0.11 |
| **Daily cost of hospital stay** | 3878±3452 | 3690±2858 | 0.56 |
| **Daily discretionary cost of ICU stay** | 1822±2466 | 2271±2523 | 0.08 |

Abbreviation List: APACHE II = Acute Physiology and Chronic Health Evaluation II; GCS = Glasgow Coma Scale; DNRCC-Arrest = Do-not-resuscitate Comfort Care Arrest; DNR = Do-not-resuscitate; ICU = medical intensive care unit

The statistical association between two categorical variables is examined using Chi-squared test.

The statistical association between a categorical variable and a continuous variable is examined using Student’s t-test.

a “Admission delay” means that the time between hospital admission and ICU admission was not zero.

b “Cared for by only one intensivist” means that the patient was cared for by only one intensivist during his/her ICU stay.

c “Floor” means that the patient was admitted to other departments before admitting to ICU.
